# Exploring the Landscape of Biomarkers in Spinal Cord Injury

**DOI:** 10.46292/sci24-00076

**Published:** 2025-06-19

**Authors:** Paulina S. Scheuren, Bethany R. Kondiles, Angela R. Filous, Ona E. Bloom, Diana S.-L. Chow, Edelle C. Field-Fote, Patrick Freund, James D. Guest, Brian K. Kwon, Nikos Kyritsis, Chris Leptak, Monica A. Perez, Matthew Szapacs, Christopher R. West, Keith Tansey, Jane T.C. Hsieh, Linda Jones

**Affiliations:** 1 International Collaboration on Repair Discoveries, University of British Columbia, Vancouver, BC, Canada; 2 Department of Anesthesiology, Pharmacology, and Therapeutics, Faculty of Medicine, Vancouver, BC, Canada; 3 Department of Zoology, University of British Columbia, Vancouver, BC, Canada; 4 Department of Neurology, Spinal Cord Injury Division, College of Medicine, The Ohio State University, Wexner Medical Center, Columbus, Ohio; 5 Neuroscience Research Institute, The Ohio State University, Columbus, Ohio; 6 Belford Center for Spinal Cord Injury, The Ohio State University, Wexner Medical Center, Columbus, Ohio; 7 Departments of Physical Medicine and Rehabilitation and Molecular Medicine, Donald and Barbara Zucker School of Medicine at Hofstra/Northwell, Manhasset, New York; 8 The Feinstein Institutes of Medical Research, Northwell Health, Manhasset, New York; 9 Department of Pharmacological and Pharmaceutical Sciences, College of Pharmacy, University of Houston, Houston, Texas; 10 Crawford Research Institute, Shepherd Center, Atlanta, Georgia; 11 Division of Physical Therapy, Emory University School of Medicine, Atlanta, Georgia; 12 Program in Applied Physiology, Georgia Institute of Technology, Atlanta, Georgia; 13 Spinal Cord Injury Center, Balgrist University Hospital, University of Zurich, Zurich, Switzerland; 14 The Miami Project to Cure Paralysis, Miller School of Medicine, The University of Miami, Miami, Florida; 15 Department of Neurological Surgery, Miller School of Medicine, The University of Miami, Miami, Florida; 16 Department of Orthopaedics, University of British Columbia, Vancouver, BC, Canada; 17 Weill Institute for Neurosciences, Brain and Spinal Injury Center, University of California, San Francisco, San Francisco, California; 18 Department of Neurological Surgery, University of California, San Francisco, San Francisco, California; 19 Greenleaf Health, Washington, DC; 20 Shirley Ryan AbilityLab, Chicago, Illinois; 21 Department of Physical Medicine and Rehabilitation, Northwestern University, Chicago, Illinois; 22 Edward Hines Jr., VA Hospital, Hines, Illinois; 23 AbbVie, Pennsylvania; 24 Centre for Chronic Disease Prevention and Management, Southern Medical Program, University of British Columbia Okanagan, Kelowna, BC, Canada; 25 United States Department of Veterans Affairs, G.V. (Sonny) Montgomery VA Medical Center, Jackson, Mississippi; 26 Department of Neurosurgery, University of Mississippi Medical Center, Jackson, Mississippi; 27 Wings for Life, Salzburg, Austria; 28 Thomas Jefferson University, Philadelphia, Pennsylvania

**Keywords:** biomarkers, participant stratification, precision medicine, prediction, prognosis, spinal cord injury clinical trials, treatment response

## Abstract

Despite considerable progress in spinal cord injury (SCI) research, there remains a pressing need for interventions that effectively restore neurological function after injury beyond that which occurs spontaneously. A major steppingstone towards the development of effective therapies for SCI is the ability to accurately predict recovery and identify individuals who are most likely to respond to intervention. Currently, the International Standards for Neurological Classification of Spinal Cord Injury (ISNCSCI) remains the primary tool for assessing neurological impairment after injury. However, based on the inherent limitations of the ISNCSCI exam, accurate and sensitive biomarkers are required. Understanding the role of biomarkers in SCI is crucial for improving diagnosis, prognosis, and treatment strategies. In 2024, the Spinal Cord Outcome Partnership Endeavour (SCOPE) sponsored a precourse at the American Spinal Injuries Association (ASIA) meeting. The international panel discussed the scope, utility, and application of biomarkers in SCI clinical trials and clinical practice. This article summarizes key insights from this discussion, highlighting the value of various types of biomarkers, ranging from molecular and cellular markers to those reflecting neural circuits, systems, and movement. We also summarize the context of using different types of biomarkers and their application in research versus clinical practice. While there are currently no FDAqualified SCI biomarkers, the development of reliable biomarkers holds the potential to accelerate the pace of discovery and enable more precise approaches to treatment.

## Introduction

**Figure 1.**
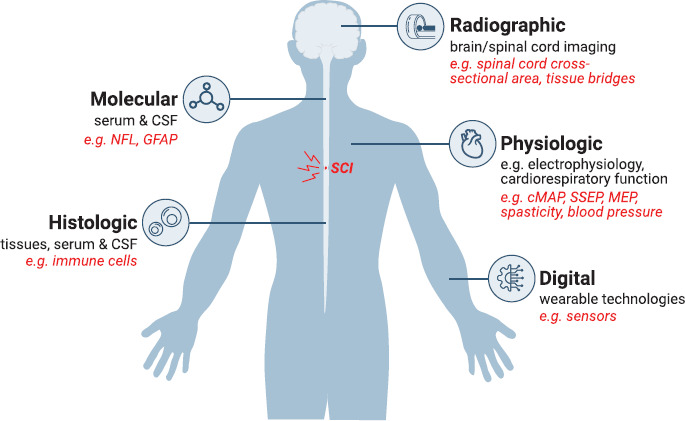
Biomarkers in spinal cord injury (SCI). Biomarkers can range from molecular (biophysical properties measured in biological samples such as blood serum and/or cerebrospinal fluid [CSF]) to histologic (biochemical or molecular alteration of cells, tissues, and fluids), radiographic (brain and spinal cord imaging studies), physiologic (measurement of body processes), and digital (wearable technologies such as movement sensors. They can also include other biomarker types such as physiologic. Potential biomarker candidates for SCI are highlighted in red for each category. cMAP = compound motor action potential; GFAP = glial fibrillary protein; MEP = motor evoked potential; NFL = neurofilament light; SSEP = somatosensory evoked potential.

A biomarker is a defined characteristic that is measured as an indicator of a normal biological processes, pathogenic processes, or biological responses to an exposure or intervention, including therapeutic interventions.^1^ Different classes of biomarkers exist, ranging from molecular to histological, radiographic, physiological, or digital (**Figure 1**),^1^ and they have revolutionized clinical practice across various medical fields.^2^ For instance, blood glucose is used as a diagnostic biomarker to identify individuals with type 2 diabetes mellitus,^3^ and breakthroughs in the field of cystic fibrosis led to sweat chloride being classified as a diagnostic and pharmacodynamic biomarker.^4^ From a regulatory perspective, biomarkers represent vital components of the drug development process, and understanding the context of use is essential.^1^ Beyond regulatory considerations, biomarkers have value in many different areas that are important for managing care, evaluating risk, monitoring therapy, and providing necessary education. Biomarkers can be used to measure a disease process by (1) confirming the presence of a disease (diagnostic biomarker), (2) estimating the likelihood of disease progression/recurrence (prognostic biomarker), and (3) indicating the potential for developing primary disease or secondary conditions (susceptibility/risk biomarker). Other biomarker uses include measures of response to intervention by (4) identifying an individual's likelihood to respond to intervention (predictive), (5) demonstrating the occurrence of a biological response to intervention (pharmacodynamic/surrogate endpoint biomarker), and (6) identifying adverse events to intervention (safety biomarker). Biomarkers that (7) assess disease status over time (monitoring biomarker) can be used to both measure disease and response to intervention.^1^

Unlike other areas of neurotrauma (e.g., traumatic brain injury, for which glial fibrillary acidic protein [GFAP] has been recently established as a biomarker),^5^ there is no US Food and Drug Administration (FDA)–qualified biomarker for injury status, severity, or prognosis in spinal cord injury (SCI). Currently, the gold standard to determine the extent of neurological impairment after injury is the International Standards for Neurological Classification of Spinal Cord Injury (ISNCSCI).^6^ While the ISNCSCI describes an internationally standardized examination to characterize injury level and severity and assign an American Spinal Injury Association Impairment Scale (AIS) grade, it was not designed as a prediction tool,^7^ and it is subject to specific challenges. A major challenge is the variability in the extent of spontaneous recovery that individuals experience even with the same baseline AIS grade.^8^ Such variability is considerable in those with incomplete injuries, but it is also prevalent in complete injuries. This heterogeneity makes it difficult to predict recovery after injury, particularly when the ISNCSCI is performed very early after injury.^8^,^9^ Moreover, the categorical nature of the AIS grading system lacks the sensitivity to predict recovery in individuals with very high or low grades of impairments, particularly in the absence of covariates.^9^–^12^ For example, individuals with AIS A can present with different recovery patterns ranging from marginal to good.^10^ Moreover, while the prognosis of ambulation is often unfavorable in individuals with AIS A, a small percentage may achieve independent walking.^11^–^13^ Conversely, ambulation is favorable in individuals with AIS D, yet some never achieve independent walking.^12^,^14^ Beyond AIS A and D, the ability to predict walking outcomes for those with AIS B and C is currently not much better than chance.^15^ The lack of a robust quantitative early biomarker obscures clinical decision-making, and the variability in spontaneous recovery makes it difficult in clinical trials to distinguish recovery that may be induced by a particular intervention versus recovery that is naturally occurring.^16^ Given the limitations of the ISNCSCI exam, there is a need for biomarkers that can accurately predict neurological recovery after injury, improve participant stratification for clinical trials (e.g., identify individuals who are more likely to respond to intervention), and detect responses to an intervention. Several promising candidates have already been recognized, and ongoing efforts may allow the identification of valuable biomarkers of SCI across different levels of resolution. The following sections will discuss the scope, utility, and application of biomarkers in human traumatic SCI.

## Exploring Biomarkers in SCI at Different Levels of Resolution

### Biofluid-based biomarkers: Molecules and cells

Beyond characterizing the initial extent of functional neurological impairment, identifying the molecular biomarkers involved in recovery after SCI is essential for adequate prognosis. The closest we can get to quantifying molecules and cells that accumulate in vivo and indicate a cascade of pathophysiological events (i.e., primary and secondary injury mechanisms) after human SCI is through the investigation of biofluids such as serum and cerebrospinal fluid (CSF).^17^ A promising line of research suggests several proteins that are sensitive to disease severity and predict neurological outcomes after traumatic SCI. Particular focus has been directed towards detecting elevated levels of neurofilament light chain (NFL), a marker of axonal injury, and GFAP, an astroglial protein indicative of structural damage to astrocytes.^18^–^21^ CSF levels of NFL and GFAP have been identified as useful diagnostic and prognostic biomarkers in the field of neurotrauma including acute SCI.^18^,^19^,^22^–^24^ Other structural proteins (i.e., tau, S100β) and inflammatory cytokines (e.g., interleukin 6, interleukin 8, monocyte chemotactic protein-1) derived from CSF 24 hours after injury are related to injury severity and AIS conversion.^18^ Ideally, a biomarker is closely associated with the target tissue. However, while CSF markers may better reflect CNS injury, practical challenges—namely, the requirement to acquire CSF—make more accessible blood-based samples more clinically accessible and translatable.

A recent study identified strong correlations between CSF and serum levels of NFL and GFAP in acute SCI.^19^ Like CSF markers, serum NFL and GFAP objectively distinguish between injury severity and predict AIS conversion,^19^–^21^ indicating their utility to act as useful prognostic and predictive markers and possible tools to improve stratification for SCI clinical trials. Serum markers allow more refined evaluation of injury severity, even within a population of individuals with the same clinical AIS grade, allowing for improved prognostication.^19^,^20^ A recent study also explored the potential of routine serological markers in predicting neurological recovery after SCI,^25^ highlighting an even more accessible approach to biomarker discovery. Although including serological markers did not improve the baseline prediction model, further stratification by acute motor scores improved prediction accuracy.^25^ The search for molecular and cellular biomarkers of SCI with prognostic and predictive value is particularly challenging in the context of timing (i.e., injury onset [prehospital], early acute, subacute, and chronic phases). Unlike CSF, serum NFL and GFAP have optimal performance at predicting outcome when measured at 48 to 72 hours,^19^ indicating minor differences between the use of CSF versus serum markers for participant stratification for SCI clinical trials. Even though much focus is directed towards predicting recovery at acute phases, sensorimotor recovery may still occur after weeks to months after injury. A recent study adopting an untargeted proteomics analysis found emerging biomarker candidates (i.e., enrichment of proteins involved in inflammation, coagulation, and lipid metabolism) in subacute SCI.^26^

The application of molecular biomarkers in SCI clinical trials not only allows the characterization of important response predictors but also provides valuable information pertaining to the actual response to a given intervention (i.e., target engagement). Recent clinical trials have adopted this approach, such as the phase II/III Riluzole in Spinal Cord Injury Study (RISCIS) trial.^27^ Pharmacokinetic and pharmacodynamic (PK/PD) analyses of riluzole revealed a PK/PD correlation model, with reduced plasma levels of phosphorylated neurofilament heavy chain (p-NFH) in response to treatment.^28^ Similarly, the AXER-204 trial—investigating the safety and efficacy of Nogo decoy therapy in SCI—identified differently expressed proteins involved in “axon guidance” and “gliogenesis” in the treated versus placebo group.^29^

There are several reasons to explore the systemic immune system as a source of potential biomarkers in acute or chronic SCI.^30^ These range from detecting susceptibility/risk of infections, monitoring acute intraspinal inflammation, and identifying risks for specific secondary complications. The bulk of understanding regarding immune system activation following SCI has come from studies in experimental, preclinical animal models, more often than not, involving rodents.^31^–^34^ In humans, two seminal postmortem studies have demonstrated that immune cells infiltrate and persist in the human spinal cord after injury.^35^,^36^ As direct evaluation of intraspinal inflammation in vivo is currently difficult in a clinical setting, most of what we know in humans is derived from studies investigating the systemic immune system that is readily accessible through the collection of CSF and blood samples.^18^–^20^,^37^–^42^ Prospective analyses of white blood cells revealed temporal and cell type-specific changes in monocytes, neutrophils, and lymphocytes (T- and B-cells).^37^ Other studies suggest that infections during the first year after injury may correlate inversely with functional recovery.^38^ Cellular analysis of CSF revealed increased erythrocytes and leukocytes after severe spinal cord injury (AIS A-B), particularly at early phases.^18^ Several independent studies have demonstrated the value of early white blood cell fluctuations as a prognostic marker of recovery after acute SCI.^39^–^41^ Moreover, recent work has also highlighted specific gene co-expression modules (gene signatures) in white blood cells that correlate with SCI severity, providing prognostic accuracy for both complete and incomplete injuries.^42^ These modules reflect immune cell subtype activity and offer insights into the peripheral immune response's potential as a diagnostic tool. By leveraging these transcriptomic modules alongside clinical data, researchers aim to refine participant stratification and tailor treatment approaches, underscoring the promise of immune profiling to improve SCI management and long-term outcomes.^41^,^42^ It has become clear that systemic immune cells may serve as potential targets to reduce inflammation and valuable candidate biomarkers in SCI. Not only are they clinically feasible to measure, but they are also modifiable (e.g., responsive to intervention),^43^ and many drugs that target the immune system have already been approved for other indications.^44^,^45^ Challenges remain, such as the need for standardized timepoints to collect samples, participant heterogeneity, the effect of comorbidities (e.g., diabetes), as well as other covariates (e.g., hormonal levels, medications, rehabilitation) that may influence immune cell function. Biomarker assessments in traumatic brain injury seek to overcome some of these challenges while developing a commercially available instrument to acquire samples and determine concentrations of GFAP.^46^

### Neurological circuits and systems: Imaging and electrophysiology

Biomarkers are not restricted to single cells or molecules detected in serum or CSF but can extend to other bodily systems and circuits. Both conventional and advanced quantitative magnetic resonance imaging (MRI) metrics represent promising techniques to serve as potential biomarkers.^47^,^48^ On a macrostructural level, characterizing intramedullary lesion parameters, such as intramedullary lesion length, hemorrhage, maximal cord compression, cross-sectional area, and mid-sagittal tissue bridges, provide insight into the degree of structural cord damage after injury.^47^–^49^ Advanced quantitative approaches that allow investigation of microstructural parameters in the brain and spinal cord (e.g., myelination, water content, iron concentration, morphometric measures) have also been shown to be reproducible across sites,^50^,^51^ an important prerequisite for the planning of multicenter clinical trials. Evidence from mono- and multicenter studies demonstrates the value of early preservation of spinal tissue bridges in predicting neurological recovery after SCI.^52^–^58^ These findings highlight the potential of conventional T2-weighted MRI scans as valuable prognostic and predictive biomarkers in SCI, supporting the concept that a certain degree of spared spinal cord tissue is needed for improved recovery after injury.^59^ The use of neuroimaging markers in SCI clinical trials was adopted in the recently completed Nogo Inhibition in Spinal Cord Injury (NISCI) trial, demonstrating larger tissue bridges in individuals with motor-incomplete compared to motor-complete injuries during the subacute phase.^60^ These findings highlight the utility of neuroimaging markers to further investigate factors influencing therapeutic effects in SCI clinical trials. Advancements in imaging technology may allow for its future use as an indicator of response to intervention.

In addition to structural MRI techniques, electrophysiological outcomes can provide functional evidence of preserved neuronal pathways after SCI.^61^–^63^ Several SCI studies have employed classical nerve conduction studies, such as compound motor action potentials (cMAPs) that are defined as the sum of all motor unit action potentials in the muscle obtained by electrical nerve stimulation.^64^ Pathological ulnar cMAP amplitudes are related to worse recovery of upper extremity function in individuals with cervical SCI,^63^,^65^,^66^ reflecting intramedullary motor neuron damage. Moreover, cMAP amplitudes are related to injury severity and early ulnar cMAP has been shown to improve SCI prognosis.^63^,^66^ Furthermore, the integrity of spinal pathways can be assessed using electroencephalographic recordings in response to electrical nerve stimulation (i.e., somatosensory evoked potentials [SSEPs]). Loss of early SSEPs, indicating diminished integrity of dorsal column pathways, is related to poor recovery after SCI.^63^,^67^–^70^ These findings demonstrate the prognostic value of electrophysiology as an objective, complementary tool to clinical investigations after acute SCI.

In addition to the assessment of ascending pathways, motor evoked potential (MEP) recordings provide information regarding the integrity of descending corticospinal pathways after injury.^71^,^72^ MEPs from proximal and distal muscles are recorded using electromyography in response to transcranial magnetic stimulation or electrical stimulation of descending pathways. Sparing of corticospinal pathways (i.e., spared MEPs) has been related to segmental motor recovery after SCI.^73^–^75^ In recent studies, residual descending motor pathways (i.e., spared MEPs) were related to the presence of spasticity below the level of injury in individuals with motor complete SCI.^76^,^77^ In other words, individuals with severe injuries that exhibit spasticity remained “anatomically incomplete.” Moreover, individuals with spasticity and preserved MEPs showed improvements in motor scores measured by the ISNCSCI exam and showed AIS conversion after subacute motor complete SCI.^78^ These findings highlight that the presence of spasticity, as a proxy of preserved corticospinal pathways, might represent a biomarker to predict recovery after SCI. The development of objective and portable systems to measure spasticity using kinematic-based inertial measurement units (IMUs)^79^ may facilitate the implementation of spasticity outcomes in SCI clinical trials.

### Other systems: Examples of cardiorespiratory biomarkers

Recovery of autonomic nervous system function is among the highest priorities for individuals living with SCI.^80^ While understudied compared to other systems (e.g., motor), the long-term dysfunction of these systems has a devastating impact on quality of life and independence and is a common cause of morbidity and mortality.^81^,^82^ This highlights the need for biomarkers of cardiorespiratory function and their inclusion in SCI clinical trials. Possible candidates include cardiac biomarkers, blood pressure, and respiratory function (i.e., forced vital capacity). Both heart size and resting blood pressure are directly related to the integrity of descending projections from the brainstem to sympathetic preganglionic neurons in the spinal cord.^83^ The value of assessing left ventricle size in individuals with cervical SCI was highlighted in recent studies demonstrating reduced cardiac function from 3 to 6 months post injury.^84^ Moreover, blood pressure measurements are easy to conduct in a clinical setting, and higher mean arterial blood pressure at early timepoints is associated with improved recovery after SCI. Another example is the assessment of forced vital capacity, which is related to injury severity^85^ and may be a potential marker of exercise capacity after SCI. These studies highlight the potential use of cardiorespiratory biomarkers to identify individuals at risk of developing cardiac and/or respiratory dysfunction that would most benefit from early intervention.

## Application of Biomarkers in SCI Clinical Trials and Precision Medicine

Biomarkers can play distinct roles in research and clinical practice, informing both clinical trials and planning of clinical care. In SCI research, the focus remains on determining whether treatments work, while other fields (e.g., oncology) have advanced to adopting stratified approaches,^86^ identifying which individuals respond best to specific treatments. In clinical practice, therapists tailor rehabilitation to individual patients to achieve optimal outcomes. In clinical trials, however, the classical randomized controlled trial approach requires standardized parameters across all participants within an assigned group. In recent years, SCI clinical trials have adopted adaptive study designs.^27^,^29^,^87^–^89^ The advantage of an adaptive trial design is that it allows for prespecified interim analyses to adjust sample size that can optimize trial outcomes.^87^,^90^ Incorporating biomarkers into such adaptive designs (e.g., to identify those who are likely to be responders vs. nonresponders) can enhance the ability to accurately interpret the outcome of interventional trials. This approach is sound in theory, but practical limitations may arise as implementing robust biomarkers into clinical trials often requires specialized, costly equipment (which puts a premium on biomarkers that can be readily and reliably measured). Additionally, careful consideration of how specific biomarkers are integrated into various trial designs is essential.

Biomarkers can also be used to increase the specificity of care and clinical decision-making for specific subgroups of individuals with SCI (i.e., determining when to use which interventions and for what purpose). Healthcare professionals often seek tools to aid in diagnosis, prognosis, and prediction of recovery after SCI to determine appropriate therapeutic strategies.^91^,^92^ This often involves the use of clinical outcome assessments,^93^ which may be somewhat similar to but different from biomarkers,^1^ highlighting the importance of standardized terminology across stakeholders. As with clinical outcome assessments, biomarkers share comparable pitfalls, requiring precision in defining purpose, and must follow standardized protocols to be meaningful and inform clinical practice.

The clinical and research utility of a biomarker depends on the ability of the biomarker to predict the construct of interest and the biomarker having advantages over other available metrics that are used to predict outcomes. Currently, clinical prediction rules that are based on clinical measures, such as presence/absence of volitional movement (e.g., the ISNCSCI exam), are routinely used for planning of care and deciding on the most appropriate interventions. Many of the current clinical prediction rules are based on movement information that is already routinely collected such as the ISNCSCI exam.^15^,^94^–^97^ Other clinical prediction rules include a combination of measures that are routinely used and measures that are common in the clinical setting.^98^

If a proposed biomarker does not represent an improvement over clinical prediction rules when it comes to making predictions about outcomes, then it must have some other advantage over clinical prediction rules. These advantages could include being more efficient to acquire the measurement or being applicable for times when it is not possible to acquire the measures needed for clinical prediction rules. The value of a biomarker that does not represent an improvement or advantage over clinical prediction rules is questionable, and for that reason comparison to clinical prediction rules is an important part of establishing the clinical and research utility of a new biomarker.

## Pathway to Biomarker Development: Considerations for SCI

The successful implementation of biomarkers relies heavily on a detailed understanding of natural history information for a disorder. SCI is fortunate in this regard, having various international registries that have developed extensive natural history data.^99^ Establishing normative values and natural progressions of biomarkers enables the determination of endpoints for treatment, providing concrete goals and timelines for regulatory consideration. When establishing evidence for a biomarker's use, it is advised to engage regulatory bodies early on to plan and establish clear timelines, metrics, and goals.^100^ Researchers and healthcare professionals should utilize available infrastructure, resources,^100^ and precedents to ensure they are meeting standards for data management, protocols, and methods of measurement. Adherence to best practices and robust evidence increases confidence in a biomarker's utility, specificity, and applications.

Biomarkers need to be reliable, replicable, and assessed using approved, validated analytical methods, which can be deployed for widespread use.^1^,^101^–^103^ Successful development of novel therapeutic targets for SCI may be facilitated by the development of a large precision medicine toolbox that reflects a spectrum of potential biomarkers. Novel approaches such as the use of wearable technologies would permit ongoing assessments,^104^ potentially detecting early signs of intervention efficacy prior to an individual's clinical assessments. The integration of bedside testing may permit real-time biomarker assessment at acute timepoints after injury, avenues that have already been explored in other areas of neurotrauma.^46^ A precision medicine toolbox for SCI may include biomarkers at different levels of resolution (i.e., multimodal biomarkers) that have proven useful in the field of neurotrauma (e.g., traumatic brain injury).^105^,^106^ Given the complexity of central nervous system disorders including SCI, the relatively small population, heterogeneity of injury, and limited statistical power pose ongoing challenges for the field. Collaborative efforts across institutions to standardize sample collection and storage (e.g., biobanking) could help address these limitations, particularly when adopting multivariate, multimodal approaches to biomarker discovery. Furthermore, established SCI biobanks may not only allow further exploration of known markers (e.g., GFAP), but also novel biomarker discovery and possible identification of new targets. The ability to integrate multimodal biomarkers that reflect discrete yet potentially overlapping processes after injury may help determine individualized precision treatment plans and predict treatment response. Despite its potential, such an approach has yet to be validated in SCI.

## Conclusion

The process of discovering and developing biomarkers requires the coordination of multiple stakeholders to provide evidence demonstrating both analytical validity and clinical utility for an intended context of use.^101^ The growing emphasis on precision medicine has heightened demand in identifying translational biomarkers (i.e., conserved across species) that indicate toxicity, target engagement, and pharmacological activity and enable more efficient clinical trials through improved participant selection and stratification.^101^ Given the substantial time and financial resources needed to advance a biomarker from development to qualification, forming consortia that include industry partners, academic institutions, and disease foundations represents the most effective long-term strategy.^101^ Striving to establish such a collaborative effort in the field of SCI would not only accelerate biomarker discovery, development, and qualification, but it would also help us achieve our ultimate goal of treating the right patient with the right intervention at the right time.
